# Ultra high-field SWI of the substantia nigra at 7T: reliability and consistency of the swallow-tail sign

**DOI:** 10.1186/s12883-017-0975-2

**Published:** 2017-10-26

**Authors:** Manuel A. Schmidt, Tobias Engelhorn, Franz Marxreiter, Juergen Winkler, Stefan Lang, Stephan Kloska, Philipp Goelitz, Arnd Doerfler

**Affiliations:** 1 Department of Neuroradiology, University Hospital Erlangen-Nuremberg, Schwabachanlage 6, 91054 Erlangen, Germany; 2 Department of Molecular Neurology, University Hospital Erlangen-Nuremberg, Schwabachanlage 6, 91054 Erlangen, Germany

**Keywords:** Nigrosome 1, Parkinson’s disease, Swallow-tail sign, SWI, Ultra high-field MRI, 7 tesla

## Abstract

**Background:**

The loss of the swallow-tail sign of the substantia nigra has been proposed for diagnosis of Parkinson’s disease. Aim was to evaluate, if the sign occurs consistently in healthy subjects and if it can be reliably detected with high-resolution 7T susceptibility weighted imaging (SWI).

**Methods:**

Thirteen healthy adults received SWI at 7T. 3 neuroradiologists, who were blinded to patients’ diagnosis, independently classified subjects regarding the swallow-tail sign to be present or absent. Accuracy, positive and negative predictive values (PPV and NPV) as well as inter- and intra-rater reliability and internal consistency were analyzed.

**Results:**

The sign could be detected in 81% of the cases in consensus reading. Accuracy to detect the sign compared to the consensus was 100, 77 and 96% for the three readers with PPV reader 1/2/3 = 1/0.45/0.83 and NPV = 1/1/1. Inter-rater reliability was excellent (inter-class correlation coefficient = 0.844, alpha = 0.871). Intra-rater reliability was good to excellent (reader 1 R/L = 0.625/0.786; reader 2 = 0.7/0.64; reader 3 = 0.9/1).

**Conclusion:**

The swallow-tail sign can be reliably detected. However, our data suggest its occurrence is not consistent in healthy subjects. It may be possible that one reason is an individually variable molecular organization of nigrosome 1 so that it does not return a uniform signal in SWI.

## Background

Great effort has been taken into finding imaging based biomarkers to facilitate diagnosis of Parkinson’s disease (PD). Diffusion tensor imaging (DTI) showed promising results in differentiating PD from healthy controls [1], however results from subsequent studies were mixed and heterogeneous so that the stability and validity of DTI derived measures for diagnosis of PD has been questioned [2].

Susceptibility mapping of the substantia nigra (SN) has been performed using ultra high field MRI and showed an increased susceptibility of the pars compacta (SNc) reflecting increased iron deposition in PD [3]. As a consequence, the image impression of the dorsolateral SN, which is similar to the split tail of a swallow in oblique-axial susceptibility weighted imaging (SWI), is lost in PD. The underlying anatomical structure of the swallow-tail configuration of the dorsal SN in healthy individuals is nigrosome 1. It represents the largest of 5 distinct clusters of dopaminergic cells of the SNc that have been identified [4]. In healthy individuals, the ovoid area of nigrosome 1 returns a high signal on high-resolution SWI and has been visualized at 7T [5] as well as on 3 T [6] in good accordance to histopathological specimen. Since nigrosome 1 is a histological concept, the term dorsolateral nigral hyperintensity (DNH) has been suggested [7]. The specific shape of the SN in healthy individuals arises from the hyperintense signal of nigrosome 1 and the hypointense signal returned by the surrounding tissue of the SN and the medial lemniscus. Subsequently, the loss of the swallow-tail appearance of the substantia nigra has been proposed as a diagnostic sign for the detection of PD, recently, by introducing the swallow-tail sign and its loss in PD [8].

In PD, degeneration of nigrosome 1 already begins at an early stage of the disease [9]. Thus, an imaging based sign such as the swallow-tail sign is of greatest interest regarding clinical practice. However, to be used as a diagnostic sign in clinical reading, the sign should occur consistently in healthy subjects and very high sensitivity, specificity and diagnostic accuracy is of utmost importance. Given the sign to be subtle even under ideal conditions - i.e. high spatial resolution; no motion artifacts, which is difficult to achieve in PD patients - aim of our study was to investigate the appearance of the swallow-tail sign in healthy individuals using ultra high-field SWI at a magnetic field strength of 7T. We examined, whether the sign is continuously present in healthy individuals, if it can be reliably detected by experienced neuroradiologists and if it’s absence can thus be used as an imaging marker for PD.

## Methods

### Subjects

We included 13 healthy subjects (mean age +/− SD = 46.7 y +/− 12.5; 5 females and 8 males) that received high resolution SWI. Subjects showed no signs nor had a history of neurological disease and severe neurological conditions such as stroke, inflammatory CNS diseases and intracranial masses were excluded. Especially, subjects showed no early signs of Parkinson’s disease such as anosmia, rapid eye movement, sleep behavior disorders or autonomic dysfunction and had no family history of PD. The Clinical Investigation Ethics Committee of the University of Erlangen-Nuremberg approved the protocol of this prospective study and the research was conducted in accordance with the Declaration of Helsinki. All participants gave written informed consent prior to all measurements.

### Imaging protocol

We used a 7T ultra high-field scanner (Magnetom Terra, Siemens Healthineers, Erlangen, Germany) with a gradient field strength of up to 80 mT/m with a slewrate of 200 T/m/s. For signal reception, a 32-channel receive array head coil (Nova Medical, Wilmington, MA, USA) was used.

Axial high resolution SWI was performed with 0.8 mm isotropic resolution using a 3D gradient echo sequence (TR = 27 ms, TE = 15 ms, FoV = 172 × 230 mm^2^). Susceptibility weighting was achieved by combining the magnitude and the filtered phase image.

For exclusion of severe neurological conditions and for anatomical orientation a high-resolution 3D MP2RAGE (Magnetization Prepared 2 Rapid Acquisition Gradient Echoes) [10] was obtained (0.8 mm isotropic resolution, TR = 4530 ms, TE = 1.92 ms, TI1 = 700 ms, TI2 = 2700 ms, FoV = 226 × 240 mm^2^).

### Image analysis and statistics

**Fig. 1:**
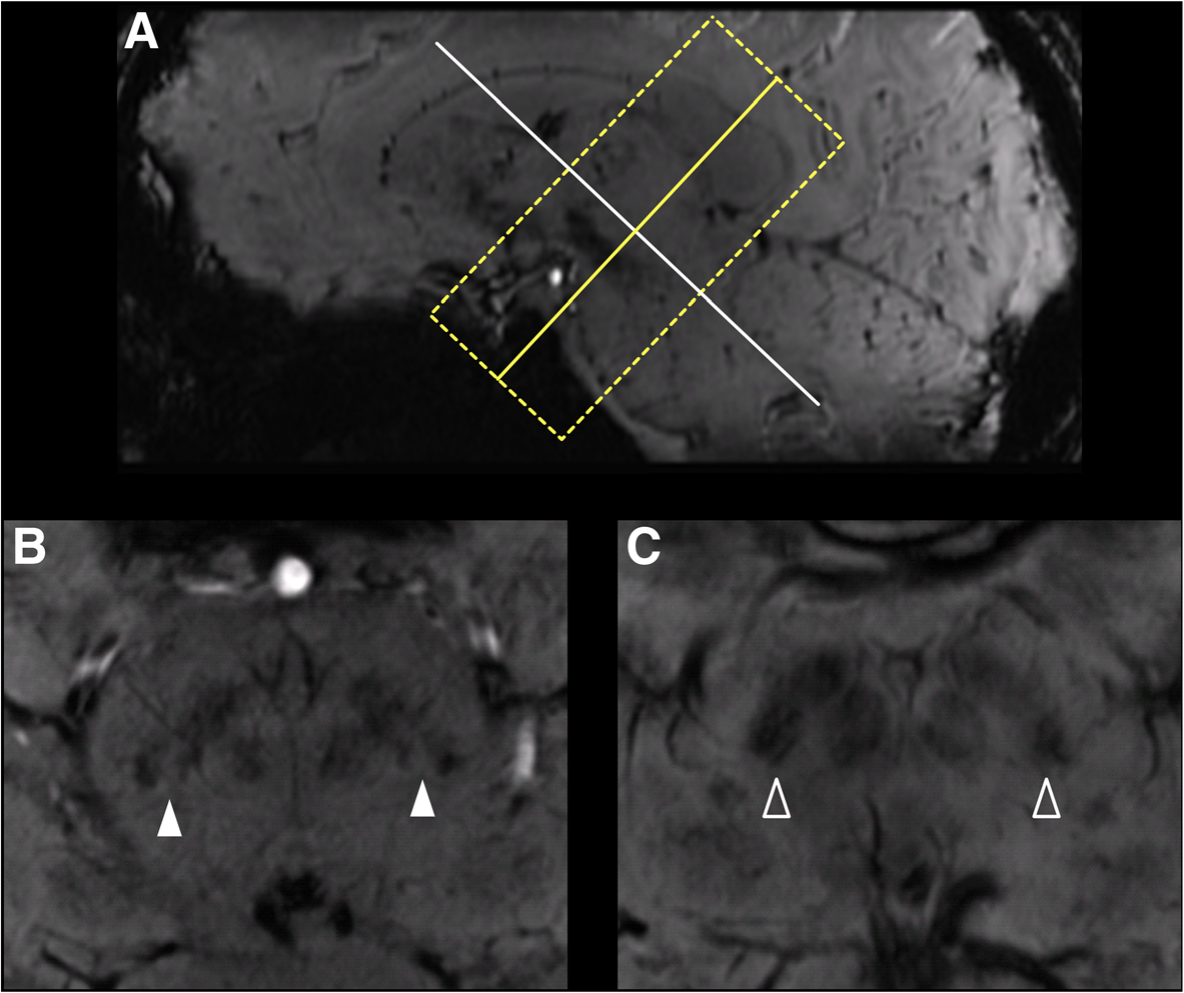
**a** Oblique-axial 1 mm MPR depicting the SN in two healthy subjects. Different slices for demonstration purposes. **b** Nigrosome 1 and consequently the swallow-tail sign can be readily visualized (filled arrow). **c** Absent swallow-tail sign (arrow)

Oblique-axial images were reconstructed perpendicular to the long axis of the midbrain in thin slice data resulting in 50 contiguous, paraaxial slices of 0.8 mm slice thickness. Three experienced neuroradiologists with 14, 12 and 11 years of experience independently read the images and classified subjects regarding the swallow-tail appearance of the dorsolateral SN to be present or absent for both sides individually (Fig. 1). Readers were blinded to the clinical diagnosis, i.e. they were not aware of the fact that the study group only included healthy subjects. Each reader repeated reading after 4 weeks. Consensus reading was performed for definitive classification after the second individual rating has been completed. Accuracy, PPV (positive predictive value), NPV (negative predictive value) as well as inter- and intra-rater reliability and internal consistency (Cronbach’s alpha) were the outcome measures analyzed.

All statistical analyses were performed using SPSS version 19.0 (SPSS, Inc., Chicago, IL, USA).

## Results

**Fig. 2:**
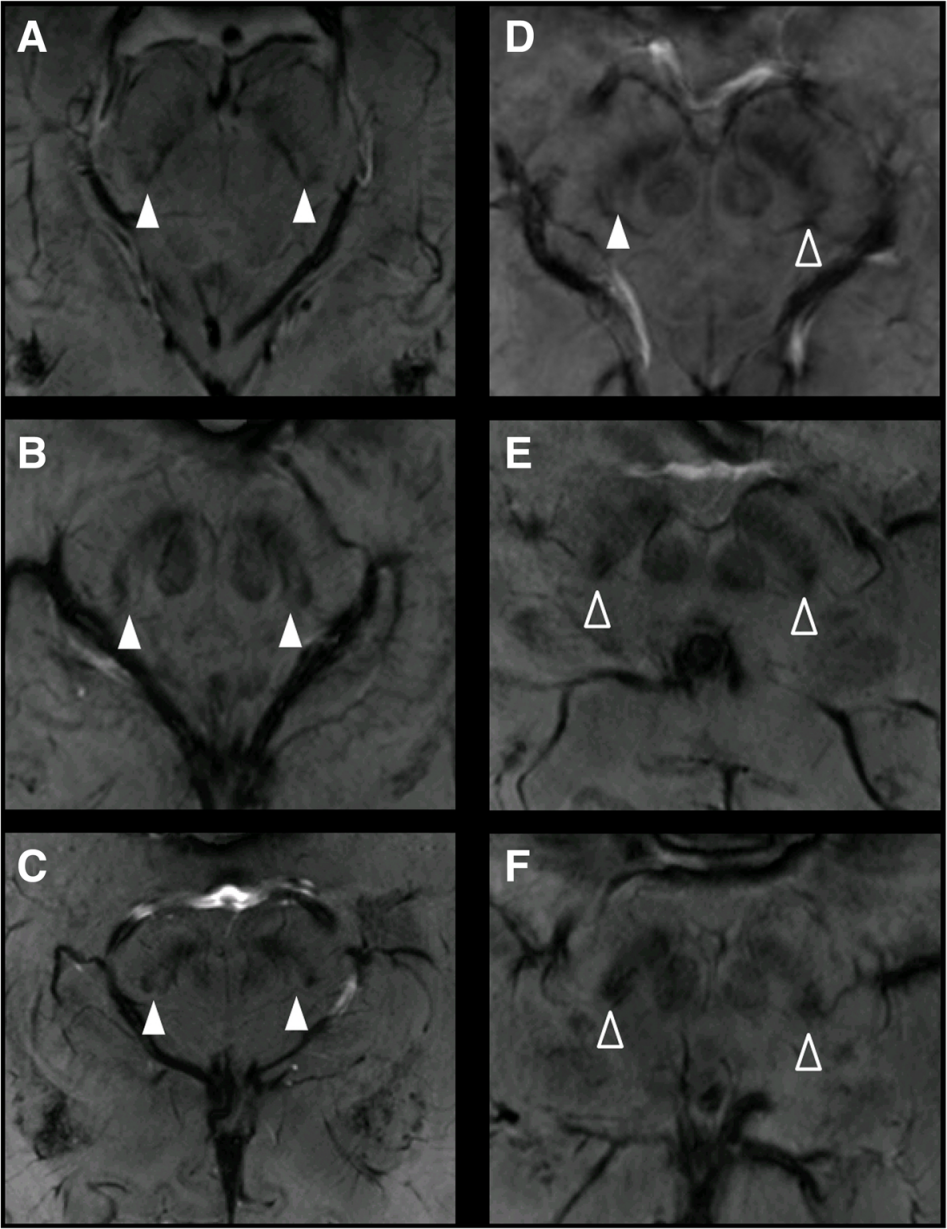
Inconsistent occurrence of the swallow-tail sign in healthy subjects. **a**-**c** Clearly definable swallow-tail of the dorsolateral SNc. **d** The swallow-tail can only be identified on the right side (filled arrow). **e**-**f** No swallow-tail sign can be detected (arrows)

In 81% of the cases (21 out of 26, each side individually) a swallow tail appearance of the dorsolateral SN could be identified in consensus reading (Table 1, Fig. 2) resulting in a false-positive rate of 19% (5/26). Accuracy against the consensus read was calculated as follows: sum of correctly classified absent and correctly classified present sign divided through the sum of correctly and falsely classified absent sign and correctly and falsely classified present sign, always corresponding to the results of consensus reading. Accuracy was 100% for reader 1, 77% for reader 2 and 96% for reader 3 in the first read. In the second read, accuracy increased for 2 readers: 77% to 100% for reader 2 and 96% to 100% for reader 3.

**Table 1:** Interpretation of the swallow-tail sign by 3 readers, y = swallow tail-sign present, n = swallow-tail absent

Patient # R/L	reader 1	reader 2	reader 3	consensus
1	y/y	y/y	y/y	y/y
2	y/y	y/y	y/y	y/y
3	n/n	y/y	y/y	y/y
4	y/y	y/y	y/y	y/y
5	y/y	y/y	y/y	y/y
6	n/y	n/y	n/y	n/y
7	y/y	y/y	y/y	y/y
8	n/n	y/y	y/y	y/y
9	n/n	n/n	n/n	n/n
10	n/n	n/n	n/n	n/n
11	y/y	y/y	y/y	y/y
12	y/y	y/y	y/y	y/y
13	n/n	y/y	n/y	y/y

PPV was 1 for reader 1, 0.45 for reader 2 and 0.83 for reader 3. NPV was 1 for all 3 readers. Results are summarized in Table 2.

**Table 2:** Presence of the swallow-tail in healthy subjects. Accuracy against the consensus read as gold standard

*n* = 13, healthy subjects	reader 1	reader 2	reader 3
positive predictive value	100%	45%	83%
negative predictive value	100%	100%	100%
accuracy	100%	77%	96%

Inter-rater reliability was excellent for all 3 readers with an inter-class correlation coefficient = 0.844 and Cronbach’s alpha = 0.871.

Regarding comparison of the two reads with the second read taking place 4 weeks after the initial read, intra-rater reliability was good to excellent for the 3 readers. For reader 1 intra-reader reliability was good regarding both sides separately (R/L = 0.625/0.786). For reader 2 intra-rater reliability was also good (R/L = 0.7/0.64). For reader 3 intra-rater reliability of both reads was excellent (R/L = 0.9/1).

## Discussion

A large study including 148 patients with PD, multiple system atrophy and progressive supranuclear palsy showed that visual assessment of dorsolateral nigral hyperintensity may serve as a simple diagnostic imaging marker for parkinsonian disorders. This study included 42 healthy controls, with DNH being absent in one case [7]. A recent meta-analysis of 10 studies also suggests that the swallow-tail sign is of high diagnostic value for the differentiation of PD from controls [11].

**Fig. 3:**
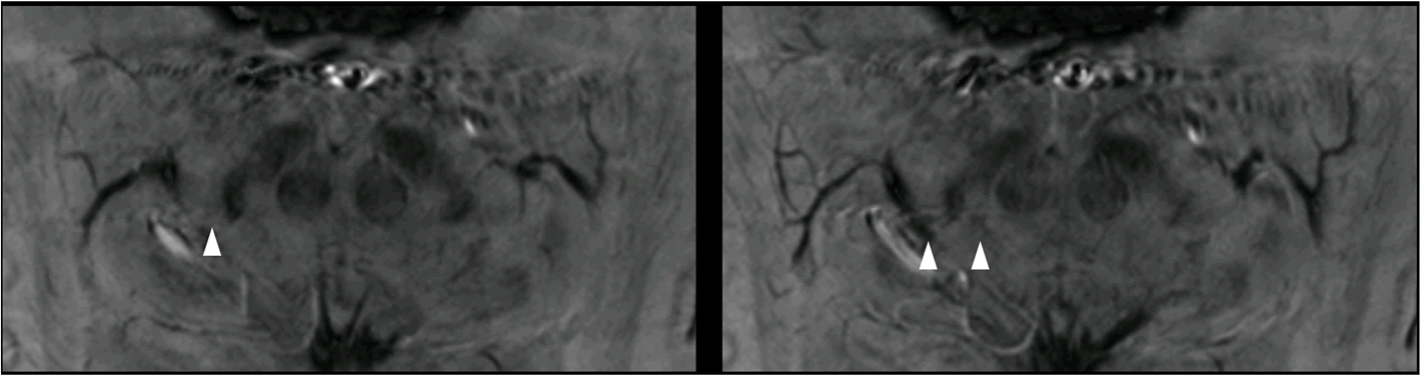
Healthy subject. Pulsation artifacts of the superior cerebelli artery could potentially disguise the swallow-tail sign (despite being recognizable in this case) leading to misclassification

In the images of the healthy subjects we evaluated, DNH and the typical swallow-tail shape of the dorsolateral SNc could be identified in only 81% (21/26, both sides separately) of the cases by three experienced neuroradiologists in consensus reading. In 2 healthy subjects, the swallow-tail sign was absent bilaterally and in 1 subject, the sign was absent on the left side (Fig. 2). In earlier reports, SWI at 7T and at 3 T was evaluated and the swallow-tail sign was present in 95% [6, 8] up to 100% [5, 12] of healthy subjects. Our study group comprised 13 healthy subjects which is comparable to the number of healthy controls in 3 of the former reports. All scans were of diagnostic quality. Especially the scans of the subjects with an absent swallow-tail sign were free from artifacts due to motion from breathing or pulsation. Even slight head motion may contribute to a blurrier image impression making DNH and thus the swallow-tail sign indefinable. Moreover, pulsation artifacts as phase-encoded motion artifacts could make the detection of DNH impossible (Fig. 3).

In the setting of the present study, readers were blinded to the clinical diagnosis, i.e. not aware of the fact that the study group only comprised healthy subjects. Regarding translation into a clinical setting, this implies that healthy subjects could be misclassified and be suspected to show nigral degeneration. NPV was 100% for all 3 readers, i.e. all readers correctly identified the 3 patients with an absent swallow-tail sign. Consensus reading took place after all 3 readers had performed the first two reads independently. Since all statistic measures were calculated in relation to the results of the consensus reading, this means that all readers independently identified the 3 patients with an absent swallow-tail sign correctly. Actually, for no individual there was a clinically suspected diagnosis of PD, which normally presents with unilateral symptoms in early stages of the disease, nor did any individual show preclinical symptoms of PD. Especially in cases where the swallow-tail sign is absent unilaterally, this may mislead to an imaging based suspected diagnosis of Parkinson’s disease and result in further diagnostic tests and radiation exposure (DaTSCAN) when the sign is applied in clinical reading.

Our results suggest that a swallow-tail appearance of the SNc may be absent in healthy subjects due to individual anatomic variants and that nigrosome 1 does not uniformly and necessarily lead to a swallow-tail appearance of the dorsolateral SN on SWI imaging. False-positive results have been described at a similar frequency before [6] when SWI was performed at 3 T and the relatively high rate was attributed to age effects as it was proposed that normal ageing may lead to changes in nigrosome 1 leading to an absent swallow-tail sign. In contrast to this previous study, in our stud group, the individuals with an absent swallow-tail sign were relatively young (41, 46 and 59 years of age), indicating a “physiological” absence of the typical swallow-tail configuration of the SN in a proportion of subjects without nigral degeneration of any cause.

False-positive results have previously also been attributed to limited spatial resolution and lower signal-to-noise and contrast-to-noise ratios that are achievable at a magnetic field strength of 3 T in a routinely reasonable scan time, so new methods like quantitative susceptibility mapping mask (QSM-mask) weighted magnitude imaging have been proposed to provide improved visualization of dorsolateral nigral hyperintensity regarding 3 T imaging [13].

Moreover, the possibility of undiagnosed PD has been quoted for an absent swallow-tail sign in a proportion of healthy subjects. Regarding the prevalence of PD in industrialized countries is generally estimated at 0.3% in the entire population and in the range of 1% in people over 60 years of age [14] to 2–3% in patients over 65 years of age [15], the probability of an undiagnosed, preclinical PD has, however, to be considered rather low. Our cohort comprised relatively young subjects (mean age = 46.7 y). Assuming a 10-year prodromal period, the reported prevalence of prodromal PD is only 0.5% at age 55 [16] and can be estimated even lower in our cohort, given the young mean age. Furthermore, there is a report that 15% of patients with drug-induced parkinsonism (DIP) showed an absent swallow-tail sign in SWI acquired at a magnetic field strength of 3 T [17]. In this study, the number of false positive results, i.e. absent dorsolateral nigral hyperintensity in healthy subjects was also 15%, which is in line with our false-positive rate of 19%. Interestingly, the DIP patients in this study received ^18^F–FP-CIT PET, which revealed normal DAT binding, excluding the possibility of subclinical drug-exacerbated parkinsonism.

This implies that an absent swallow-tail configuration in some subjects may not be the result of nigral degeneration with increased iron deposition. Instead differences in the microstructural organization of nigrosome 1 may be the reason of a hypointense signal in SWI so that nigrosome 1 cannot be visualized. This notion is supported by a very recent study examining the neuromelanin-related T2* contrast in the SN [18] which suggests that metal-bound, intra- and extracellular neuromelanin macromolecules may cause a hypointense signal of nigrosome 1 in T2*/SWI imaging, especially at high magnetic field strengths. Additionally, nano-secondary ion mass spectrometry was used in a recent investigation to show differences in the chemical composition of individual neuromelanin granules of the SN of healthy subjects [19]. As a consequence, interindividually different amounts and different molecular compositions of neuromelanin in the SNc may lead to a not fully hyperintense signal of nigrosome 1 in SWI and thus annihilate the swallow-tail configuration in a proportion of healthy subjects.

Our results of PPV and NPV match those of previous reports [8]. Inter-rater reliability was excellent in our study and is also in line with earlier reports [6, 20].

Of interest, accuracy to correctly detect the sign increased with the second read for two readers reaching 100%. This implies that even experienced neuroradiologists need to get familiar with the image impression when using the swallow-tail sign to correctly classify patients. Neuroradiologists should thus be actively trained to get familiar with the microanatomy of the SN to be able to memorize an engram of the histological appearance of nigrosome 1 when applying the swallow-tail sign in their reports.

Our study has limitations. It is retrospective study with a relatively small number of subjects, and a prospective study with more participants will be needed to confirm our findings. Despite the possibility of preclinical PD can be considered rather low, because the participants were relatively young and showed no preclinical symptoms such as anosmia, rapid eye movement, sleep behavior disorders or autonomic dysfunction, we cannot fully exclude preclinical PD. This is especially important, as absence of DNH on high-field SWI may potentially identify prodromal degenerative parkinsonism [21]. A longitudinal study may therefore be needed.

## Conclusion

The swallow-tail sign can be reliably detected using high-resolution SWI at 7T, however its occurrence is not consistent as the sign may be absent in healthy adults due to an interindividually different molecular organization of nigrosome 1 so that it does not return a uniform signal in SWI. Even an experienced reader should be well trained to confidently use the swallow-tail sign.

## Abbreviations

CNS: Central nervous system
DNH: Dorsolateral nigral hyperintensity
DTI: Diffusion tensor imaging
FoV: Field of view
MPR: Multi-planar reformation
NPV: Negative predictive value
PD: Parkinson’s disease
PPV: Positive predictive value
SN: Substantia nigra
SNc: Substantia nigra pars compacta
SWI: Susceptibility weighted imaging
TE: Echo time
TR: Repetition time

## References

[1] Vaillancourt DE, Spraker MB, Prodoehl J, Abraham I, Corcos DM, Zhou XJ, Comella CL, Little DM (2009). High-resolution diffusion tensor imaging in the substantia nigra of de novo Parkinson disease. Neurology.

[2] Schwarz ST, Abaei M, Gontu V, Morgan PS, Bajaj N, Auer DP (2013). Diffusion tensor imaging of nigral degeneration in Parkinson's disease: a region-of-interest and voxel-based study at 3 T and systematic review with meta-analysis. NeuroImage Clin.

[3] Lotfipour AK, Wharton S, Schwarz ST, Gontu V, Schafer A, Peters AM, Bowtell RW, Auer DP, Gowland PA, Bajaj NP (2012). High resolution magnetic susceptibility mapping of the substantia nigra in Parkinson's disease. J Magn Reson Imaging.

[4] Damier P, Hirsch EC, Agid Y, Graybiel AM (1999). The substantia nigra of the human brain. I. Nigrosomes and the nigral matrix, a compartmental organization based on calbindin D(28K) immunohistochemistry. Brain.

[5] Blazejewska AI, Schwarz ST, Pitiot A, Stephenson MC, Lowe J, Bajaj N, Bowtell RW, Auer DP, Gowland PA (2013). Visualization of nigrosome 1 and its loss in PD: pathoanatomical correlation and in vivo 7 T MRI. Neurology.

[6] Noh Y, Sung YH, Lee J, Kim EY (2015). Nigrosome 1 detection at 3T MRI for the diagnosis of early-stage idiopathic Parkinson disease: assessment of diagnostic accuracy and agreement on imaging asymmetry and clinical laterality. AJNR Am J Neuroradiol.

[7] Reiter E, Mueller C, Pinter B, Krismer F, Scherfler C, Esterhammer R, Kremser C, Schocke M, Wenning GK, Poewe W (2015). Dorsolateral nigral hyperintensity on 3.0T susceptibility-weighted imaging in neurodegenerative parkinsonism. Mov Disord.

[8] Schwarz ST, Afzal M, Morgan PS, Bajaj N, Gowland PA, Auer DP (2014). The ‘swallow tail’ appearance of the healthy nigrosome - a new accurate test of Parkinson’s disease: a case-control and retrospective cross-sectional MRI study at 3T. PLoS One.

[9] Damier P, Hirsch EC, Agid Y, Graybiel AM (1999). The substantia nigra of the human brain. II. Patterns of loss of dopamine-containing neurons in Parkinson’s disease. Brain.

[10] Marques JP, Kober T, Krueger G, van der Zwaag W, Van de Moortele PF, Gruetter R (2010). MP2RAGE, a self bias-field corrected sequence for improved segmentation and T1-mapping at high field. NeuroImage.

[11] Mahlknecht P, Krismer F, Poewe W, Seppi K (2017). Meta-analysis of dorsolateral nigral hyperintensity on magnetic resonance imaging as a marker for Parkinson’s disease. Mov Disord.

[12] Kim JM, Jeong HJ, Bae YJ, Park SY, Kim E, Kang SY, Oh ES, Kim KJ, Jeon B, Kim SE (2016). Loss of substantia nigra hyperintensity on 7 Tesla MRI of Parkinson's disease, multiple system atrophy, and progressive supranuclear palsy. Parkinsonism Relat Disord.

[13] Nam Y, Gho SM, Kim DH, Kim EY, Lee J (2017). Imaging of nigrosome 1 in substantia nigra at 3T using multiecho susceptibility map-weighted imaging (SMWI). J Magn Reson Imaging.

[14] de Lau LM, Breteler MM (2006). Epidemiology of Parkinson’s disease. Lancet Neurol.

[15] Poewe W, Seppi K, Tanner CM, Halliday GM, Brundin P, Volkmann J, Schrag AE, Lang AE (2017). Parkinson disease. Nat Rev Dis Primers.

[16] Berg D, Postuma RB, Adler CH, Bloem BR, Chan P, Dubois B, Gasser T, Goetz CG, Halliday G, Joseph L (2015). MDS research criteria for prodromal Parkinson’s disease. Mov Disord.

[17] Sung YH, Noh Y, Lee J, Kim EY (2016). Drug-induced parkinsonism versus idiopathic Parkinson disease: utility of Nigrosome 1 with 3-T imaging. Radiology.

[18] Lee JH, Baek SY, Song Y, Lim S, Lee H, Nguyen MP, Kim EJ, Huh GY, Chun SY, Cho H (2016). The Neuromelanin-related T2* contrast in postmortem human Substantia Nigra with 7T MRI. Sci Rep.

[19] Biesemeier A, Eibl O, Eswara S, Audinot JN, Wirtz T, Pezzoli G, Zucca FA, Zecca L, Schraermeyer U (2016). Elemental mapping of Neuromelanin organelles of human Substantia Nigra: correlative ultrastructural and chemical analysis by analytical transmission electron microscopy and nano-secondary ion mass spectrometry. J Neurochem.

[20] Cosottini M, Frosini D, Pesaresi I, Costagli M, Biagi L, Ceravolo R, Bonuccelli U, Tosetti M (2014). MR imaging of the substantia nigra at 7 T enables diagnosis of Parkinson disease. Radiology.

[21] De Marzi R, Seppi K, Hogl B, Muller C, Scherfler C, Stefani A, Iranzo A, Tolosa E, Santamaria J, Gizewski E (2016). Loss of dorsolateral nigral hyperintensity on 3.0 tesla susceptibility-weighted imaging in idiopathic rapid eye movement sleep behavior disorder. Ann Neurol.

